# Mogroside V protects porcine oocytes from *in vitro* ageing by reducing oxidative stress through SIRT1 upregulation

**DOI:** 10.18632/aging.102324

**Published:** 2019-10-06

**Authors:** Junyu Nie, Lumin Sui, Huiting Zhang, Hengye Zhang, Ke Yan, Xiaogan Yang, Shengsheng Lu, Kehuan Lu, Xingwei Liang

**Affiliations:** 1 State Key Laboratory for Conservation and Utilization of Subtropical Agro-Bioresources, Guangxi University, Nanning 530004, Guangxi, China; 2 College of Animal Science and Technology, Guangxi University, Nanning 530004, Guangxi, China

**Keywords:** mogroside V, oocyte ageing *in vitro*, ROS, SIRT1, embryo development

## Abstract

Postovulatory ageing compromises oocyte quality and subsequent development in various manners. We aimed to assay the protective effects of mogroside V on porcine oocyte quality during *in vitro* ageing and explore the related causes. We observed that mogroside V can effectively maintain normal oocyte morphology and early embryo development competence after prolonged culture for 24 h. Moreover, mogroside V can markedly reduce reactive oxygen species (ROS) levels, alleviate spindle formation and chromosome alignment abnormalities, improve mitochondrial contents, adenosine triphosphate (ATP) levels and the membrane potential (ΔΨm), and reduce early apoptosis in aged oocytes. We examined the molecular changes and found that *SIRT1* expression was decreased in *in vitro* aged oocytes but was maintained by exposure to mogroside V. However, when *SIRT1* was successfully inhibited by the specific inhibitor EX-527, mogroside V could not reduce ROS levels or alleviate abnormal spindle organization and chromosome misalignment. In summary, our results demonstrated that mogroside V can alleviate the deterioration of oocyte quality during *in vitro* ageing, possibly by reducing oxidative stress through SIRT1 upregulation.

## INTRODUCTION

Postovulatory oocyte ageing is a process in which MII (metaphase II) oocytes undergo a time-dependent deterioration in quality when no fertilization takes place within the ideal time frame [1]. Furthermore, this process generates aged oocytes of deteriorated quality, with oocytes showing mitochondrial dysfunction, spindle/ chromosome abnormalities, apoptosis, and fertilization and early embryo development failure [2, 3]. Postovulatory ageing is also harmful to preimplantation and mid-term gestational development, as well as the fitness of offspring [4]. These defects may be caused by oxidative stress induced by postovulatory ageing [4]. Thus, attenuation of oxidative stress may alleviate the deterioration in oocyte quality induced by postovulatory ageing [5, 6].

*Siraitia* *grosvenorii* (Luo-han-guo, LHG) is an edible and medicinal plant primarily distributed in Guangxi, China [7]. Because of their sweetness (approximately 300-times sweeter than sucrose), low caloric value and low toxicity [8, 9], extracts made from the fruit of LHG are generally recognized as safe by the Food and Drug Administration (FDA) [4] and are used as an alternative to sucrose in many countries, such as Japan, America, Australia and New Zealand [10]. The triterpenoid compounds in LHG, which are called mogrosides, are the primary active compounds that give LHG its sweetness. Approximately 46 triterpenoid compounds, including mogrosides I, III, IV and V, have been isolated from LHG [10], and mogroside V (MV) is the most abundant form and is the most well studied [7, 11, 12].

Numerous studies have demonstrated that mogrosides have various biological activities, such as reducing blood glucose and lipid levels [13], as well as anti-diabetic [2], anti-inflammatory [14], anti-tumour and anti-carcinogenic effects [7, 11, 12]. In addition, several studies have reported that MV is a powerful antioxidant that scavenges free radicals [15]. Mogrosides, the primary component of which is MV, reduce oxidative stress in the livers of high-fat diet-induced obese mice and alloxan-induced diabetic mice [16–18]. In addition, MV reduces the production of pro-inflammatory cytokines, protecting lung tissue against acute lung injury induced by lipopolysaccharide (LPS) [19]. In RAW264.7 cells, MV reduces the increase in reactive oxygen species (ROS) levels induced by LPS and blocks the phosphorylation of AKT1 [20]. Recent studies have shown that cucurbitane triterpenoids containing MV may be potential *AMPK* activators [21]. Taken together, these findings demonstrate that mogrosides have important biological characteristics, and some of the associated mechanisms have been explored. However, the effects of mogrosides on alleviating female subfertility have yet to be elucidated.

In this study, we explored whether MV prevents porcine oocytes from *in vitro* ageing. We examined oocyte morphology, early embryo development following parthenogenetic activation, ROS levels, spindle organization, chromosome alignment, mitochondrial content, adenosine triphosphate (ATP) levels, membrane potential (ΔΨm), and early apoptosis. In addition, we also attempted to elucidate the underlying mechanisms associated with the protective effects of MV on aged oocytes. Our findings may provide a new strategy to protect against oocyte deterioration during postovulatory ageing.

## RESULTS

### Protective effects of MV on oocyte ageing *in vitro*

**Figure 1:**
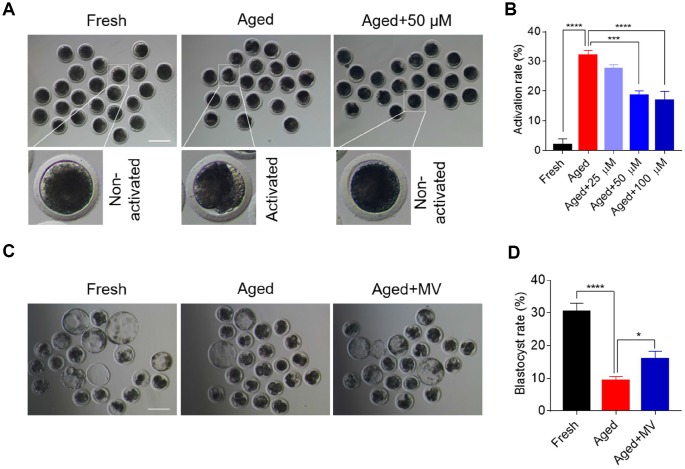
**Mogroside V alleviates the deterioration in oocyte quality during *in vitro* ageing.** After *in vitro* maturation for 44 h, oocytes that extruded the first polar body were continuously cultured *in vitro* with or without MV (25, 50 and 100 μM) for 24 h. (**A**) Views of oocytes after activation. (**B**) Activation rates of oocytes in the fresh, aged, and aged+MV groups. (**C**) Representative images of blastocysts from fresh, aged and aged+MV oocytes. (**D**) Blastocyst formation rates of fresh, aged and aged+MV oocytes. MV, mogroside V; Scale bar = 200 μm. The data are presented as the mean ± SEM of at least three independent experiments. * *P* < 0.05, *** *P* < 0.001, **** *P* < 0.0001.

MII oocytes are predisposed to activation due to a decline in quality during postovulatory ageing. Therefore, we first examined whether MV can alleviate the activation of aged oocytes by assaying the effects of different doses of MV (25, 50 and 100 μM) based on our preliminary experiments and published reports [4, 15]. As shown in Figure 1A and 1B, cleaved and fragmented oocytes were regarded as activated. The activated proportion of aged oocytes was markedly higher than that of fresh oocytes (fresh 2.0±2.00%, n=105, *VS* aged 32.1±1.65%, n=84, *P* < 0.0001), suggesting that *in vitro* ageing compromises oocyte quality. Notably, MV attenuated the activation of aged oocytes with increasing concentrations (Figure 1B). These results showed that MV reduced the activation rate of the aged oocytes from 32.1 to 18.5% at 50 μM (aged 32.1±1.65%, n=84, *VS* aged+50 μM 18.5±1.55%, n=86, *P* < 0.001) and 16.9% at 100 μM (aged 32.1±1.65%, n=84, *VS* aged+100 μM 16.9±3.03%, n=84, *P* < 0.0001) (Figure 1B). However, no significant difference in the activation rate was found between the 50 μM and 100 μM MV groups (aged+50 μM 18.5±1.55%, n=86, *VS* aged+100 μM 16.9±3.03%, n=84, *P* > 0.05, Figure 1B). Therefore, 50 μM MV was used for subsequent experiments.

We next examined early development competence to further determine whether MV may prevent oocytes from *in vitro* ageing. Accordingly, parthenogenetic activation and *in vitro* embryo culture were performed. As shown in Figure 1C and 1D, the blastocyst formation rate of aged oocytes was significantly lower than that of fresh oocytes (fresh 30.4±2.59%, n=98, *VS* aged 9.5±1.12%, n=126, *P* < 0.0001). However, MV significantly improved the blastocyst formation rate of aged oocytes from 9.5 to 16.0% (aged 9.5±1.12%, n=126, *VS* aged+MV 16.0±2.30%, n=118, *P* < 0.05, Figure 1C, 1D). Collectively, these results demonstrate that MV can alleviate oocyte quality deterioration induced by *in vitro* ageing.

### The effect of MV on ROS levels in aged oocytes

**Figure 2:**
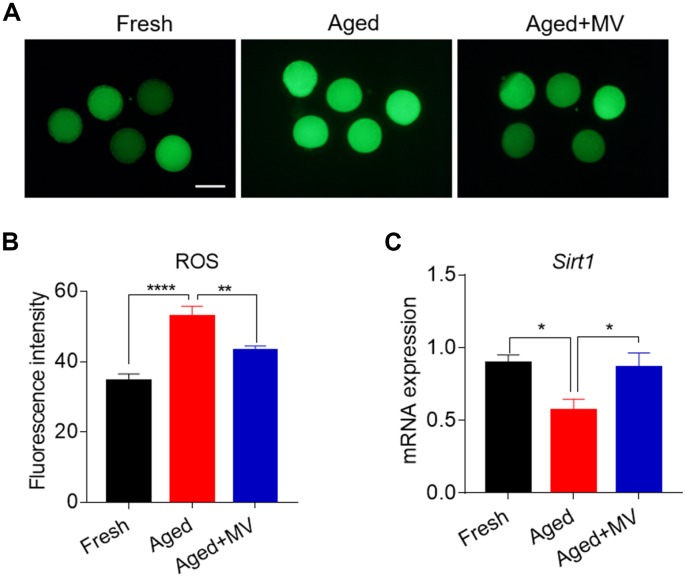
**Effects of mogroside V on the ROS level and *SIRT1* expression in oocytes during *in vitro* ageing**. After *in vitro* maturation for 44 h, oocytes that extruded the first polar body were continuously cultured *in vitro* with or without MV for 24 h. (**A**) Representative images of fresh, aged and aged+MV oocytes stained with DCFH-DA. (**B**) Quantitative analysis of ROS fluorescence intensity in fresh, aged and aged+MV oocytes. (**C**) The mRNA expression of *SIRT1* in fresh, aged and aged+MV oocytes. The data are presented as the mean ± SEM of at least three independent experiments. MV, mogroside V; Scale bar = 100 μm. * *P*< 0.05, ** *P* < 0.01, **** *P*< 0.0001.

Oxidative stress is induced by the accumulation of ROS in aged oocytes and is a primary factor adversely affecting oocyte quality. Thus, we next evaluated whether MV can alleviate oxidative stress. For this evaluation, a DCFH-DA probe was used to assay oocyte intracellular ROS levels. As shown in Figure 2A and 2B, the fluorescence intensity of DCFH-DA in aged oocytes was significantly increased compared with that observed in fresh oocytes (fresh 34.6±1.94, n=46, *VS* aged 52.9±2.93, n=48, *P* < 0.0001), suggesting that *in vitro* ageing induces oxidative stress in oocytes. Interestingly, in the presence of MV, the fluorescence intensity of DCFH-DA in aged oocytes was significantly reduced (aged 52.9±2.93, n=48, *VS* aged+MV 43.1±1.40, n=45, *P* < 0.01, Figure 2A, 2B), indicating that MV can alleviate oxidative stress.

We further examined *SIRT1* expression due to its regulation of ROS generation in aged oocytes [26]. As shown in Figure 2C, the level of *SIRT1* mRNA was significantly lower in aged oocytes than that in fresh oocytes (fresh 0.9±0.06 *VS* aged 0.6±0.08, *P* < 0.05, Figure 2C). As expected, MV exposure increased *SIRT1* mRNA levels (aged 0.6±0.08 *VS* aged+MV 0.9±0.10, *P* < 0.05, Figure 2C), demonstrating that MV can promote the expression of key genes involved in oxidative stress in aged oocytes.

### Effects of MV on reducing spindle/chromosome abnormalities in aged oocytes

**Figure 3:**
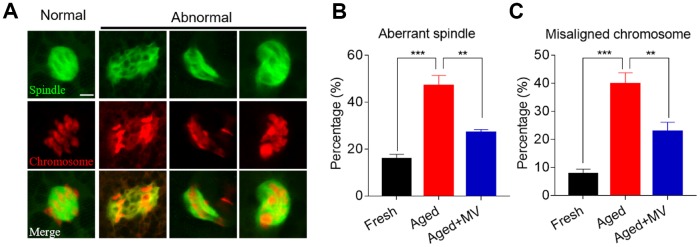
**Effects of mogroside V on spindle formation and chromosome alignment in oocytes during *in vitro* ageing.** After *in vitro* maturation for 44 h, oocytes that extruded the first polar body were continuously cultured *in vitro* with or without MV for 24 h. (**A**) Representative images of spindle morphologies and chromosome alignment in fresh, aged and aged+MV oocytes. (**B**) The percentage of aberrant spindle formation in fresh, aged and aged+MV oocytes. (**C**) The percentage of misaligned chromosomes in fresh, aged and aged+MV oocytes. The data are presented as the mean ± SEM of at least three independent experiments. MV, mogroside V; Scale bar = 7.5 μm. ** *P*<0.01, *** *P*< 0.001.

Because elevated ROS levels cause cytoskeletal defects in aged oocytes, we next explored whether MV can reverse spindle organization and chromosome alignment abnormalities by promoting a reduction in ROS levels. Oocytes were stained with anti-α-tubulin FITC antibody and PI to view spindle morphology and chromosome alignment, respectively. As shown in Figure 3A and 3B, the rate of aberrant spindle formation in aged oocytes was vastly higher than that in fresh oocytes (fresh 15.7±2.07%, n=51, *VS* aged 47.1±4.35%, n=53, *P* < 0.001), but was significantly reduced in aged oocytes treated with MV from 47.1 to 27.0% (aged 47.1±4.35%, n=53, *VS* aged+MV 27.0±1.29%, n=48, *P* < 0.01). Similarly, as shown in Figure 3A and 3C, the misaligned chromosome rate of aged oocytes was markedly higher than that of fresh oocytes (fresh 7.7±1.69%, n=51, *VS* aged 39.8±3.99%, n=53, *P* < 0.001), but MV significantly reduced the misaligned chromosome rate of aged oocytes from 39.8 to 22.7% (aged 39.8±3.99%, n=53, *VS* aged+MV 22.7±3.37%, n=48, *P* < 0.01). The above results showed that MV can alleviate spindle organization and chromosome alignment abnormalities in *in vitro* aged oocytes.

### Effects of MV on promoting mitochondrial function in aged oocytes

**Figure 4:**
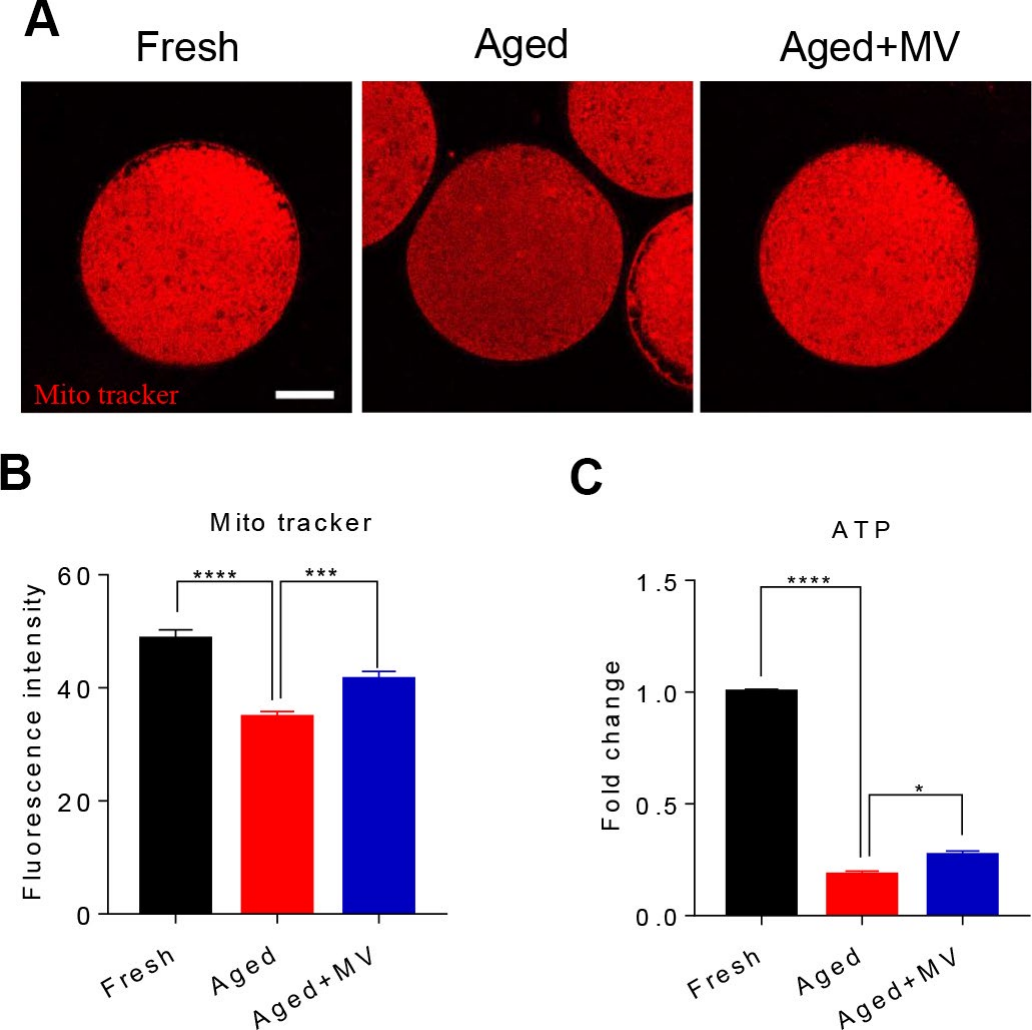
**Effects of mogroside V on mitochondrial and ATP contents in oocytes during *in vitro* ageing.** After *in vitro* maturation for 44 h, oocytes that extruded the first polar body were continuously cultured *in vitro* with or without MV for 24 h. (**A**) Representative images of fresh, aged and aged+MV oocytes stained with MitoTracker™ Orange CMTMRos. (**B**) Quantitative analysis of the mitochondrial content. (**C**) Quantitative analysis of the ATP content. The data are presented as the mean ± SEM of at least three independent experiments. MV, mogroside V; Scale bar = 50 μm. * *P* < 0.05, *** *P* < 0.001, **** *P* < 0.0001.

We assessed mitochondrial content with a Mito Tracker probe and found that the fluorescence intensity of Mito Tracker in aged oocytes was significantly reduced compared to that in fresh oocytes (fresh 48.6±1.64, n=68, *VS* aged 34.8±1.07, n=73, *P* < 0.0001, Figure 4A, 4B), but MV significantly increased the fluorescence intensity of Mito Tracker (aged 34.8±1.07, n=73, *VS* aged+MV 41.5±1.48, n=75, *P* < 0.001, Figure 4A, 4B). Because mitochondrial function primarily generates ATP as the energy required for cell activities, we next examined the intracellular ATP content. As shown in Figure 4C, the ATP content of aged oocytes was dramatically lower than that of fresh oocytes (fresh 1.0±0.01, n=3 *VS* aged 0.2±0.02, n=3, *P* < 0.0001, Figure 4C). However, after MV supplementation, the ATP content significantly increased compared with that observed in aged oocytes (aged 0.2±0.02, n=3, *VS* aged+MV 0.3±0.02, n=3, *P* < 0.05, Figure 4C).

**Figure 5:**
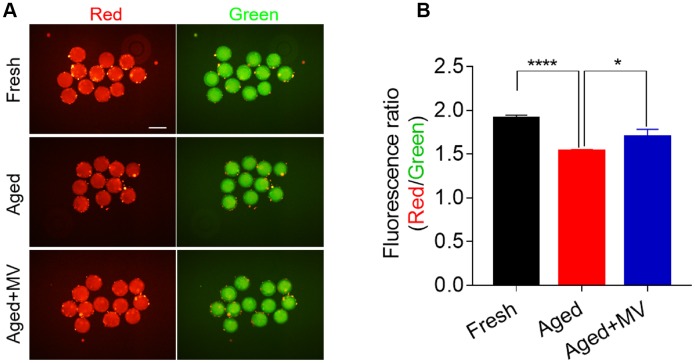
**Effect of mogroside V on the mitochondrial membrane potential in oocytes during *in vitro* ageing.** After *in vitro* maturation for 44 h, oocytes that extruded the first polar body were continuously cultured *in vitro* with or without MV for 24 h. (**A**) Representative images of fresh, aged and aged+MV oocytes stained with JC-1. (**B**) Quantitative analysis of JC-1 fluorescence intensity. The data are presented as the mean ± SEM of at least three independent experiments. Mogroside V, MV; Scale bar = 200 μm. * *P*< 0.05, **** *P*< 0.0001.

Mitochondrial membrane potential (ΔΨm) is another indicator reflecting mitochondrial function. Therefore, we evaluated the ΔΨm to further determine the mitochondrial function of oocytes. Oocytes were stained with JC-1, and the ratio of red/green fluorescence was used to determine the membrane potential. As shown in Figure 5, the red/green ratio in aged oocytes was significantly lower than that in fresh oocytes (fresh 1.9±0.03, n=60, *VS* aged 1.5±0.02, n=60, *P* < 0.0001, Figure 5A, 5B). In contrast, MV significantly increased the red/green ratio (aged1.5±0.02, n=60, *VS* aged+MV 1.7±0.09, n=52, *P* < 0.05, Figure 5A, 5B). Collectively, our data showed that MV exposure increased the mitochondrial content, ATP levels and the ΔΨm, suggesting that MV exposure enhances the activity and function of mitochondria in aged oocytes.

### The effect of MV on inhibiting the early apoptosis of aged oocytes

**Figure 6:**
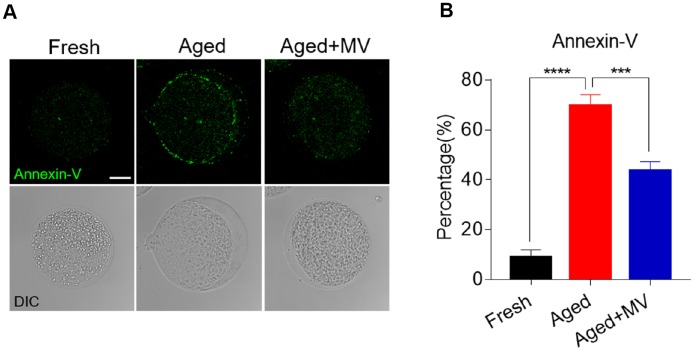
**Effect of mogroside V on early apoptosis in oocytes during *in vitro* ageing.** After *in vitro* maturation for 44 h, oocytes that extruded the first polar body were continuously cultured *in vitro* with or without MV for 24 h. (**A**) Representative images of fresh, aged and aged+MV oocytes stained with Annexin-V. (**B**) Quantitative analysis of Annexin-V fluorescence intensity. The data are presented as the mean ± SEM of at least three independent experiments. Mogroside V, MV; Scale bar = 50 μm. *** *P* < 0.001, **** *P* < 0.0001.

As oxidative stress accelerates the early apoptosis of oocytes during postovulatory ageing, we next assessed apoptosis induction among oocytes using an Annexin-V probe. A notable green apoptosis signal was observed in the aged oocyte membrane (Figure 6A). The proportion of Annexin-V positivity in aged oocytes was significantly higher than that in fresh oocytes (fresh 8.8±3.15%, n=79, *VS* aged 69.8±4.42%, n=62, *P* < 0.0001, Figure 6B). After MV supplementation, as shown in Figure 6A and 6B, apoptotic signals were weakened in the membranes of aged oocytes, and the proportion of Annexin-V positivity was significantly lower than that observed in aged oocytes without MV (aged 69.8±4.42%, n=62, *VS* aged+MV 43.6±3.61%, n=62, *P* < 0.001, Figure 6A, 6B), suggesting that MV exposure inhibits early apoptosis during oocyte ageing *in vitro*.

### MV prevents oocytes from *in vitro* ageing by upregulating SIRT1 expression

**Figure 7:**
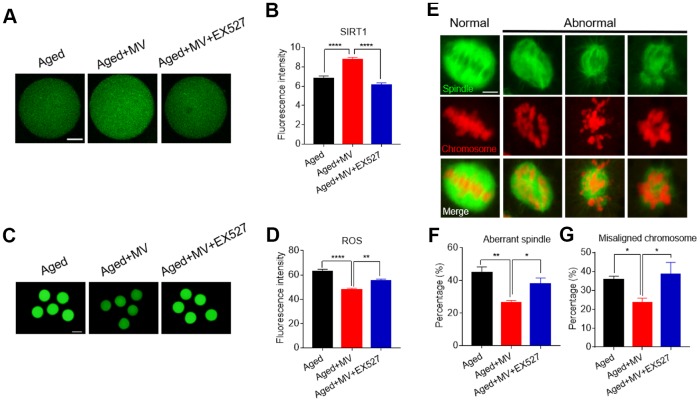
**Inhibition of SIRT1 abolishes the protective effect of mogroside V on oocytes during *in vitro* ageing.** After *in vitro* maturation for 44 h, oocytes that extruded the first polar body were continuously cultured *in vitro* with MV or MV plus the SIRT1 inhibitor EX527 for 24 h. (**A**) Representative images of aged, aged+MV and aged+MV+EX-527 oocytes stained with SIRT1 antibody. (**B**) Quantitative analysis of SIRT1 fluorescence intensity. (**C**) Representative images of aged, aged+MV and aged+MV+EX-527 oocytes stained with DCFH-DA. (**D**) Quantitative analysis of ROS fluorescence intensity. (**E**) Representative images of spindle morphologies and chromosome alignment in aged, aged+MV and aged+MV+EX-527 oocytes. (**F**) Quantitative analysis of the abnormal spindle formation rate. (**G**) Quantitative analysis of the misaligned chromosome rate. The data are presented as the mean ± SEM of at least three independent experiments. Mogroside V, MV; Scale bar, A = 50 μm, C = 200 μm, E = 5 μm. * *P* < 0.05, ** *P* <0.01, **** *P* < 0.0001.

The results presented in Figure 2 show that MV promoted the expression of *SIRT1*, which was associated with a reduction in ROS levels in aged oocytes, although whether *SIRT1* controls ROS generation remains to be unelucidated. To test this possibility, the SIRT1-specific inhibitor EX-527 together with MV was added to the medium (aged+MV+EX527) during *in vitro* ageing. At the end of treatment, the oocytes were stained with SIRT1 antibody to measure the SIRT1 protein level. As shown in Figure 7A and 7B, aged+MV oocytes had higher SIRT1 protein levels than those of aged oocytes, which is consistent with the mRNA levels shown in Figure 2C. Together, these data confirmed that MV can enhance SIRT1 expression in aged oocytes. As expected, aged+MV+EX527 oocytes had a markedly lower SIRT1 protein level than that of aged+MV oocytes (aged+MV 8.7±0.23, n=104, *VS* aged+MV+EX527 6.1±0.20, n=105, *P* < 0.0001, Figure 7A, 7B). Because SIRT1 upregulation is related to a reduction in the ROS level in aged oocytes, we next examined the ROS levels in the oocytes. In contrast to the SIRT1 expression level, we found that aged+MV oocytes had lower ROS levels than aged oocytes (aged 62.7±2.09, n=41, VS aged+MV 47.7±1.37, n=44, *P* < 0.0001, Figure 7C, 7D), but aged+MV+EX527 oocytes had higher ROS levels than aged+MV oocytes (aged+MV 47.7±1.37, n=44, VS aged+MV+EX527 55.0±1.72, n=38, *P* < 0.01, Figure 7C, 7D), and no difference was found between aged and aged+MV+EX527 oocytes. Moreover, as shown in Figure 7E and 7F, the aberrant spindle rate of aged+MV oocytes was lower than that of aged oocytes (aged 44.7±3.54%, n=62, VS aged+MV 26.4±1.33%, n=68, *P* < 0.01), but the aberrant spindle rate of aged+MV+EX527 oocytes was higher than that of aged+MV oocytes (aged+MV 26.4±1.33%, n=68, VS aged+MV+EX527 38.0±3.61%, n=50, *P* < 0.05). The same trend found for the aberrant spindle rate was also observed for the misaligned chromosome rate (aged 35.9±1.71%, n=62, VS aged+MV 23.6±2.47%, n=68, *P* < 0.05; aged+MV 23.6±2.47%, n=68, VS aged+MV+EX527 38.4±6.43%, n=50, *P* < 0.05) (Figure 7E–7G). Taken together, these data demonstrated that downregulation of SIRT1 expression leads to ROS accumulation and related damage in aged oocytes, which can be alleviated by MV to prevent oocyte quality deterioration.

## DISCUSSION

In this study, we examined the effects of MV on attenuating porcine oocyte deterioration resulting from *in vitro* ageing and attempted to elucidate the related causes. Our findings demonstrated that MV can alleviate the development of spindle/chromosome abnormalities and mitochondrial dysfunction, reduce early apoptosis and enhance the development of competence to inhibit oocyte damage during *in vitro* ageing. Numerous studies have documented that MV can potentially ameliorate metabolic disorders and treat some diseases using cultured cells or mouse models [27]. However, the effects of MV on female reproduction have remained unclear, and our findings demonstrated that MV can delay oocyte *in vitro* ageing, demonstrating a new function of MV.

We first observed that porcine oocytes exhibited a high activation rate and a low developmental capacity after culturing for a prolonged period *in vitro*. The findings are consistent with those of a previous study in which aged porcine oocytes were observed to be prone to being activated by presenting cleavage and fragments after weak electrical stimulation [28]. These phenomena indicate that oocyte quality declines during *in vitro* ageing, which was further confirmed by a subsequent reduction in the early embryonic development competence. We next attempted to elucidate the causes associated with the decline in oocyte quality and observed excessive accumulation of ROS in the aged oocytes, which can potentially lead to oxidative stress [29, 30]. Oxidative stress can damage oocytes in a variety of manners, such as disruption of spindle assembly and chromosome alignment in aged mouse and pig oocytes [26, 31], abnormalities that were consistent with our observations. In addition, excessive accumulation of ROS also impairs mitochondrial function [32, 33]. Our results showed that the mitochondrial content, the ΔΨm and ATP production were reduced in aged oocytes. Early apoptosis is often associated with mitochondrial dysfunction [34], and as expected, we observed that apoptosis was induced in aged oocytes. Overall, the findings of our lab and those of previous studies show that oxidative stress is the cause of oocyte damage, which occurs through a variety of mechanisms, during the *in vitro* ageing process [1, 25, 34, 35].

Importantly, we observed that MV can protect against the adverse effects caused by *in vitro* ageing. First, we observed that the oocyte activation rate was reduced, and the development of competence was enhanced after oocytes were exposed to MV during *in vitro* ageing. As MV is an antioxidant that can directly scavenge free radicals [36] and inhibit ROS production in the cultured cells [18, 20] and the IVM porcine oocytes [37], our results demonstrated that MV reduced ROS levels in aged oocytes. As expected, MV ameliorated the abnormal microtubule assembly and chromosomal misalignment in oocytes caused by *in vitro* ageing. In addition, MV increased mitochondrial contents, partially maintained ATP contents, and reduced early apoptosis in aged oocytes. Because MV can reduce ROS levels, its protective effects on aged oocytes may be due to it is an antioxidative ability to scavenge free radicals accumulated in oocytes during *in vitro* ageing.

*SIRT1* plays a crucial role during oogenesis by regulating energy homoeostasis, mitochondrial biogenesis, and chromatin remodelling and by protecting against oxidative stress [28, 38]. Moreover, some studies have reported that *SIRT1* participates in regulating the postovulatory ageing process of oocytes [6, 26], and that inhibition of *SIRT1* expression can alleviate the adverse effects of postovulatory oocyte ageing [31, 39]. These reports prompted us to explore whether MV acts through *SIRT1* to delay oocyte *in vitro* ageing. We first observed that *SIRT1* expression was reduced in aged oocytes, which is consistent with previous reports [6, 26, 31]. Notably, after exposure to MV, the *SIRT1* expression level was similar to that observed in fresh oocytes. Interestingly, the *SIRT1* expression level was the opposite of that of the ROS content in the fresh, aged and aged+MV groups. To determine whether *SIRT1* is the key mediator of the effects of MV, the SIRT1-specific inhibitor EX-527 was used. When SIRT1 was successfully inhibited by this inhibitor, MV treatment was ineffective at reducing ROS levels in aged oocytes. Moreover, the protective role of MV in spindle morphology and chromosome alignment was also abrogated. These results indicate that *SIRT1* is an essential regulator of MV in the protection of oocytes against the adverse effects of *in vitro* ageing. Previous studies have confirmed that upregulation of *SIRT1* expression by resveratrol or melatonin protects against oocyte deterioration during postovulatory ageing [6, 31]. Although the underlying mechanism associated with the MV-mediated promotion of *SIRT1* expression remains unclear, SIRT1 has been reported to upregulate the expression of the antioxidant enzyme MnSOD to reduce ROS levels in aged oocytes [6].

In summary, MV was shown to reverse abnormalities of the oocyte cytoskeleton, mitochondrial dysfunction, and early apoptosis by upregulating SIRT1 expression to protect against oocyte quality deterioration induced by *in vitro* ageing. Our findings reveal a new function of MV, which suggest that MV may alleviate some female reproductive disorders. In the future, comprehensive studies should be carried out to explore the effects of MV on human and animal reproduction.

## MATERIALS AND METHODS

The chemicals and reagents used in the present study were purchased from Sigma Chemical Co. (St. Louis, MO, USA), except where mentioned otherwise.

### Oocyte collection and *in vitro* maturation

Ovaries were obtained from juvenile pigs slaughtered at a local slaughterhouse and transferred to the laboratory in 0.9% saline at 32 °C. Follicular fluid from 3-8 mm antral follicles was aspirated by a syringe with an 18-gauge needle attached. As previously described [22], approximately 40-50 cumulus oocyte complexes (COCs) were cultured in 200 μl of in vitro maturation (IVM) medium, covered with mineral oil and cultured for 44 h at 38.5 °C in a 5% CO2 incubator with humidified air.

### Oocyte *in vitro* ageing and MV treatment

According to our previous description [23], the cumulus cells were removed from COCs through 0.1% hyaluronidase in PVA-TL-HEPES. The oocytes extruding the first polar body (fresh) were continuously cultured in PZM-3 medium supplemented with or without MV (aged+MV and aged, respectively) (Biopurify Phytochemicals Ltd., Chengdu, China) for 24 h. The *in vitro* ageing conditions were the same as those for IVM.

The SIRT1-specific inhibitor EX-527 was dissolved in dimethyl sulfoxide (DMSO), and the final concentration of DMSO in the medium did not exceed 0.1%. An EX-527 concentration of 20 μM was used based on a previously published report [24].

### Oocyte activation and *in vitro* culture

A weak stimulus (0.6 kV/cm, 2 direct-current pulses for 10 μs) and parthenogenetic activation (1.2 kV/cm, 2 direct-current pulses for 30 μs) were applied according to our previous report and another report [22, 25] using a BTX Elector-Cell Manipulator 2001 (BTX Inc., San Diego, CA, USA). The medium used for activation contained 0.30 M mannitol, 1.00 mM CaCl_2_·2H_2_O, 0.10 mM MgSO_4_, 0.50 mM HEPES and 0.3% (w/v) bovine serum albumin (BSA). After the operation, the embryos were immediately transferred to PZM-3 and cultured at 38.5°C in a 5% CO_2_ incubator with humidified air. Cleavage and fragments were examined after 8 h, and blastocyst formation was examined after 6 d.

### Determination of ROS levels

To determine the intracellular ROS content, oocytes were incubated for 30 min at 38.5 °C in PBS containing a 10 μM DCFH-DA fluorescent probe (Beyotime Biotechnology, Shanghai, China). The oocytes were then washed with PBS supplemented with 1% BSA. Images were captured by a confocal microscopy system (LAS - Leica TCS-SP8, Germany) with the same scanning settings among groups. Fluorescence intensity was calculated with NIH ImageJ.

### Determination of the mitochondrial content

To examine the mitochondrial content, oocytes were cultured with 200 nM orange-fluorescent dye (Mito Tracker™ Orange CMTMRos, Thermo Fisher Scientific, MA, USA) for 30 min at 38.5 °C and subsequently fixed in 4% paraformaldehyde (PFA) for 30 min at room temperature (RT). After washing several times with 0.1% PVA-PBS, the oocytes were mounted on glass slides, and images were captured under a confocal laser scanning microscope (LAS - Leica TCS-SP8, Germany). The fluorescence intensity of the oocytes was calculated with NIH ImageJ.

### Measurement of the adenosine triphosphate (ATP) content

The ATP content was measured using an ATP assay kit (Beyotime Biotechnology, Shanghai, China) according to the manufacturer’s instructions. Oocytes were washed with PBS, and twenty oocytes were pooled in one sample (three samples/group) for ATP measurement.

### JC-1 staining

To determine the mitochondrial membrane potential (ΔΨm), the oocytes were stained with JC-1 (Beyotime Biotechnology, Shanghai, China), a fluorescent probe that accumulates in mitochondria and indicates the membrane potential across the matrix membrane. Subsequently, the oocytes were incubated in 10 μM JC-1 diluted in PBS for 30 min at 38.5 °C, washed, and then imaged using a fluorescence microscope system (Nikon, Japan). The fluorescence intensity was quantified with NIH ImageJ, and the ratio of red to green fluorescence pixels was used to analyse the mitochondrial membrane potential.

### Annexin-V staining

Oocytes were stained using an Annexin-V staining kit (Beyotime Biotechnology, Shanghai, China) according to the manufacturer’s instructions. After washing twice with PBS, viable oocytes were stained with 90 μl of binding buffer containing 10 μl of Annexin-V-fluorescein isothiocyanate (FITC) for 10 min at RT in the dark. Fluorescence signals were imaged under a confocal laser scanning microscope (LAS - Leica TCS-SP8, Germany) and calculated with NIH ImageJ.

### RNA extraction, reverse transcription and RT-PCR

Approximately fifty oocytes were lysed using an RNA extraction kit (Tiangen, China). cDNA was obtained by a first strand cDNA synthesis kit (Bio-Rad, USA). In addition, RT-PCR was performed in a reaction containing 10 μl of SYBR II Mix (Takara, Japan), 0.3 μM of forward primer and reverse primer and cDNA. The following primers were used to amplify the sequence of *SIRT1*: Forward primer: ATCGTCACCAATGGTTTCCA, reverse primer: GGATCTGTGCCAATCATGAG. RT-PCR was performed on a Bio-Rad CFX 96 (USA) unit using the following programme: 1 cycle at 95 °C for 30 s and 39 cycles at 95 °C for 5 s and 60 °C for 30 s. As previously described, eGFP was added to the oocyte sample before RNA isolation [23]. The expression of eGFP was used for normalization, and relative expression levels were determined by the 2^−ΔΔCt^ method.

### Immunofluorescent staining and confocal microscopy

Oocytes were collected and washed with PBS supplemented with 0.1% PVA and then fixed with 4% PFA at RT for 30 min. The oocytes were then permeabilized with 1% Triton-100 in PBS at RT for 8–12 h and blocked with 1% BSA for 1 h at RT. After washing with 0.05% Tween-20 containing 0.1% Triton-100 in PBS, the oocytes were incubated with anti-α-tubulin FITC antibodies (Thermo Fisher Scientific, USA) at a dilution of 1:200, and an anti-SIRT1 antibody (Santa Cruz, CA, USA) at a dilution of 1:200 overnight at 4 °C. After washing in 0.05% Tween-20 containing 0.1% Triton-100 in PBS, the oocytes were stained with Alexa Fluor 488 (ZSGB-BIO, Beijing, China) secondary antibody for 1 h at RT. Oocytes were incubated in 10 μg/ml propidium iodide (PI) at RT to view the nucleus. After several washes, the oocytes were mounted on glass slides, and images were captured under a confocal laser scanning microscope (LAS - Leica TCS-SP8, Germany) with the same scanning settings.

### Statistical analysis

Data are presented as the mean ± SEM. At least three replicates were tested for each group. Statistical analyses were performed using Prism 7 software (GraphPad, San Diego, CA, USA) with analysis of variance (ANOVA) where appropriate. Fluorescence intensity was calculated by NIH ImageJ software. *P* < 0.05 indicated a statistically was significantly difference.
